# A Qualitative Study of Provider Thoughts on Implementing Pre-Exposure Prophylaxis (PrEP) in Clinical Settings to Prevent HIV Infection

**DOI:** 10.1371/journal.pone.0040603

**Published:** 2012-07-11

**Authors:** Emily A. Arnold, Patrick Hazelton, Tim Lane, Katerina A. Christopoulos, Gabriel R. Galindo, Wayne T. Steward, Stephen F. Morin

**Affiliations:** 1 Center for AIDS Prevention Studies, University of California San Francisco, San Francisco, California, United States of America; 2 HIV/AIDS Division, San Francisco General Hospital, University of California San Francisco, San Francisco, California, United States of America; Asociacion Civil Impacta Salud y Educacion, Peru

## Abstract

**Background:**

A recent clinical trial demonstrated that a daily dose tenofovir disoproxil fumarate and emtricitabrine (TDF-FTC) can reduce HIV acquisition among men who have sex with men (MSM) and transgender (TG) women by 44%, and up to 90% if taken daily. We explored how medical and service providers understand research results and plan to develop clinical protocols to prescribe, support and monitor adherence for patients on PrEP in the United States.

**Methods:**

Using referrals from our community collaborators and snowball sampling, we recruited 22 healthcare providers in San Francisco, Oakland, and Los Angeles for in-depth interviews from May-December 2011. The providers included primary care physicians seeing high numbers of MSM and TG women, HIV specialists, community health clinic providers, and public health officials. We analyzed interviews thematically to produce recommendations for setting policy around implementing PrEP. Interview topics included: assessing clinician impressions of PrEP and CDC guidance, considerations of cost, office capacity, dosing schedules, and following patients over time.

**Results:**

Little or no demand for PrEP from patients was reported at the time of the interviews. Providers did not agree on the most appropriate patients for PrEP and believed that current models of care, which do not involve routine frequent office visits, were not well suited for prescribing PrEP. Providers detailed the need to build capacity and were concerned about monitoring side effects and adherence. PrEP was seen as potentially having impact on the epidemic but providers also noted that community education campaigns needed to be tailored to effectively reach specific vulnerable populations.

**Conclusions:**

While PrEP may be a novel and clinically compelling prevention intervention for MSM and TG women, it raises a number of important implementation challenges that would need to be addressed. Nonetheless, most providers expressed optimism that they eventually could prescribe and monitor PrEP in their practice.

## Introduction

After two decades of limited biological means to prevent the transmission of HIV, the HIV prevention community was significantly reinvigorated with the announcement of results from the iPrEx trial, which showed significant evidence for prevention in the form of Preexposure prophylaxis (or PrEP)[1]. Announced in November 2010, the iPrEx trial found that in a sample of high risk MSM and TG women, a daily dose of Truvada®, a combination of two antiretrovirals, tenofovir disoproxil fumarate and emtricitabrine (TDF-FTC), could reduce the risk of transmission by 44% overall [2], [3]. This was called a “game changer” by leaders in the field. Since then, the results of several other trials using antiretroviral drugs for prophylactic purposes have been announced, including the FEM-PrEP, Partners PrEP, TDF2 and the VOICE trials [4]. Unfortunately, FEM-PrEP and VOICE were both halted early due to no significant differences between the treatment arms and placebo arms on HIV acquisition among women enrolled in them. FEM-PrEP enrolled 1951 heterosexual women in South African, Kenya, and Tanzania, and was halted in April 2011 due to a lack of significant difference between those taking a daily oral dose of TDF-FTC and the placebo arm. The VOICE Study initially discontinued only its oral tenofovir pill arm when the DSMB observed no difference in HIV incidence between that treatment arm and the placebo arm. The same absence of statistical difference was then observed two months later in the trial’s tenofovir gel arm versus placebo arm, which were also discontinued. Scientists are examining data from both halted trials to better understand adherence patterns, as well as the difference between physiology and drug uptake in women versus men, which could account for the different effects observed [4], [5]. In a more encouraging turn, the Partners PrEP study, which enrolled the seronegative partner of 4758 serodiscordant couples in Kenya and Uganda, found that those heterosexual men and women who took a daily oral dose of TDF were 62% less likely than the placebo arm to acquire HIV, while those who took a daily oral dose of TDF-FTC were 73% less likely than the placebo arm to acquire HIV [6]. Similarly, the TDF2 trial, which enrolled 1219 heterosexual men and women in Botswana and randomized them to either a daily oral dose of TDF-FTC or a placebo, found an overall protective efficacy of 62.6% for those in the treatment arm [7].

While there has been significant progress, a great deal of work remains to be done to examine how the prophylactic use of antiretroviral medications, whether taken orally or applied vaginally in the form of a gel, will be perceived by the medical community and by those in the communities most hard-hit by the epidemic in the United States. Despite the release of guidance by the Centers for Disease Control and Prevention for use of Truvada® for HIV prevention [8], and the recent approval by an expert panel at the United States Food and Drug Administration (FDA) for its prophylactic use [9], [10], many questions remain regarding implementing PrEP in clinical settings. Specifically, questions around dosing recommendations, need for medical monitoring, adherence, level of side effects, and the optimal “PrEP delivery package” have arisen [11]. More research is necessary to understand provider views on actually implementing PrEP in their clinical settings, including comfort prescribing PrEP, and considerations around building clinical protocols and office capacity to support and monitor adherence for patients on PrEP. The specific aims of our study were to better understand how medical and service providers would potentially implement PrEP in their clinical settings, and to establish the practice and clinic policies needed to package PrEP as a form of HIV prevention in the United States.

### Background

In order for PrEP to become a viable form of HIV prevention, concerns about accessing PrEP through the medical system must be addressed. It is widely acknowledged that clinical capacity as well as public health infrastructures must be strengthened in order to deliver PrEP in clinical settings, both nationally and internationally [12]. Indeed, even in the wake of clinical guidance from the Centers for Disease Control and Prevention for administering Truvada® to patients to prevent HIV acquisition, there is little agreement on the clinical protocols, not to mention public health infrastructure, community education, provider training, and clinical capacity necessary for PrEP to be implemented successfully on a national level [4], [13].

From a public health perspective, PrEP would likely be recommended for a targeted vulnerable populations as part of a more comprehensive multi-component package of HIV prevention services, which include addressing factors that may impede adherence (such as mental illness and substance use), minimizing HIV-risk behaviors, and regularly monitoring for side effects and seroconversion [13], [14], [15]. Indeed, among policy analysts there is some controversy over whether adequate public health systems exist to detect resistant strains that may emerge should PrEP be implemented without more careful monitoring and attention to adherence [16]. And while cost effectiveness studies show that targeted implementation of PrEP to high-risk MSM populations has the potential to impact the epidemic [17], [18], there are still political, economic, and ethical considerations about providing PrEP at low to no cost for those most at risk that must be explored and resolved [4], [19], [20], [21].

In this paper, we explore how providers approach managing patients who want to use PrEP as a prevention strategy. The fact that Truvada® is available only with a prescription necessitates at-risk individuals as well as public health officials to interact with medical doctors. It is essential to understand emerging clinical practices and protocols for implementing PrEP among medical providers as we begin to offer this new form of HIV prevention more widely to vulnerable populations.

## Methods

A team of researchers (EA, PH, TL, GG, and KC), including 3 social scientists, a policy analyst specializing in PrEP, and a clinician/HIV specialist, conducted in-depth interviews with 22 healthcare provider interviews in 3 cities in California (10 in San Francisco, 7 in Oakland, and 5 in Los Angeles) from May-December 2011. The project was funded as part of a collaborative HIV policy research center that pairs an academic institution responsible for conducting policy studies (UCSF) with two community partners responsible for identifying emerging issues of importance to HIV policymaking (Project Inform, San Francisco AIDS Foundation). Relying on initial referrals from our two community partners and employing snowball sampling, we purposively recruited HIV specialists, primary care physicians seeing high numbers of MSM or TG women in their practices, providers in community based and STI clinics, and public health officials in all three cities. Participants had to report monitoring patients on anti-retroviral therapy, including for post or pre-exposure propylaxis, and seeing high numbers of MSM or TG women in their practices, clinics or jurisdictions, in order to be eligible. We included providers with these characteristics because we felt that they would most likely be early adopters of providing PrEP in their clinical settings, and they might have patients that had requested PrEP. Public health officials were included in the sample because local health departments play a role in garnering support for low to no cost provision of PrEP to vulnerable populations and help implement HIV prevention public health strategies. Guided by the tenets of grounded theory, purposive sampling was conducted until we reached data saturation, and no new information was emerging in our interviews. Please see Table 1 for a breakdown of informant characteristics. Participants reported working in HIV prevention and care from 1 to 30 years, representing a range of experience. Interviews were done in person or by phone (in the case of providers who could not be scheduled when a research team was on site), and lasted approximately 45 minutes to 1 hour. Eligible participants provided verbal informed consent to participate in a one-on-one in-depth interview following a semi-structured interview guide, and received a $100.00 stipend in appreciation for their time and insights. Because the primary risk associated with this research was privacy, we explicitly sought and received approval from the Institutional Review Board to use verbal consent procedures with our informants. To preserve the anonymity of participants, all informants are referred to using pseudonyms. Interview topics for medical and service providers included: assessing general knowledge of the iPrEx trial results and PrEP, the suggested CDC guidance, considerations of cost, the capacity of primary care practices to complete necessary billing and follow-up care, dosing schedules and toxicity monitoring, and following patients regularly over long periods of time. Interviews were recorded, transcribed, and thematically analyzed to produce recommendations for setting clinical guidelines or clinic policies around implementing PrEP. All study procedures were reviewed and approved by the University of California San Francisco Institutional Review Board, the Committee on Human Research.

**Table 1:** Informant Characteristics.

Type of Informants	Sample Size (N)
Primary Care Provider (PCP)	(7)
HIV Specialist	(4)
Public Health Official	(3)
Community/STD Clinic Based Provider	(6)
Clinician Researchers	(2)
Female	(7)
Male	(15)

### Analysis

Data analysis followed a thematic analysis approach utilizing several qualitative data analysis techniques, including inductive analysis, cross-case analysis, and analytical coding of textual data [22]. Initial inductive analyses involved discovering emergent themes and patterns within the dataset to develop a preliminary project codebook [23]. From this preliminary codebook, code names and definitions evolved to match emerging data during iterative analyses of the interviews by project staff. Two research team members (EA and PH) met regularly to build coding consensus, to become familiar with participant narratives, to contextualize discrepancies, and to make coding and cross-case analysis decisions of newly uncovered themes. Memos were written to elaborate on themes derived from the data and compare across cases. Through reading and coding five common transcripts, coder agreement reached 90%, at which point the research team completed final coding of the dataset. Quotes selected for inclusion here reflect the experiences and views of participants, and were chosen based on their relevance to the research question and study aims. We used Atlas.ti software to facilitate management and analysis of qualitative data [24].

## Results

Five themes emerged during data analysis that have implications for setting clinical protocols and informing future public health programming regarding PrEP. See Table 2 for a summary of our results. The results and illustrative quotes are described in more detail below.

**Table 2:** Key Themes from Providers.

Themes	Key Quotes
There is little consensus on the target population for PrEP	*This is going to be such a limited resource, that we want to make sure that it’s not necessarily going to all the worried well.*
Current models of care are not always well suited for prescribing PrEP	*We are not used to having people that come back for check-ins on a regular basis.*
	*We wouldn’t be able to operate it if RN’s were excluded from providing [it].*
Providers need more capacity before they can prescribe PrEP	*If we wanted our medical assistants or anyone to provide PREP, they would require some counseling training.*
	*So it would require some supports, both around education, around adherence, and also financially, because we have as many uninsured as we do – we’d have to have access to meds and I’m sure it’s expensive.*
Monitoring patients on PrEP will be challenging	*I think a lot of young people tend to have less stable schedules.*
	*They might take it just 3 days before an event, if they know that they’re going to have a party or something special.*
PrEP has public health benefit	*And so only treating the positive partner isn’t going to eliminate all the infections, and so finding the right balance between treatment and PrEP I think is important to have as a target.*

### 1) Little Consensus on PrEP Target Populations

There was no real consensus about who should receive PrEP, and often accessibility to the prophylactic medication depended on where patients sought out their care, in private or public clinical settings. Most providers agreed that serodiscordant couples were seen as ideal candidates for PrEP. Providers in public clinics felt it should be only given to those at highest risk, and only if it could be at low to no cost; those in private practice said they would prescribe for any patient that wanted PrEP and whose insurance would cover the costs. One San Francisco primary care physician (PCP) in private practice felt it was appropriate to prescribe it to the “worried well,” those gay men who, even after being assessed as low risk through counseling, may still experience significant worry about their risk for HIV infection. In contrast, those at sites where Truvada® was going to be provided at low to no cost to patients felt strongly that a protocol to determine eligibility for PrEP by risk for HIV acquisition should be put in place:

*“This is going to be such a limited resource, that we want to make sure that it’s not necessarily going to all the worried well… I think some of the categories like sero-discordant couples, clearly, would be in a higher-risk category, and the rectal infection history, and PEP use in the past. And receptive unprotected anal intercourse. We’d probably figure out what the risk stratification would be, … and then what we’d consider sort of lower-risk that probably wouldn’t’ qualify for PrEP.”* (Michelle, SF provider, Community/STI Clinic).

Other physicians, particularly those who were HIV specialists and had experience treating patients who had developed resistant strains, thought that in addition to risk assessment, a provider’s assessment on who might be adherent was an important eligibility consideration.

*You kind of have to be in that sweet spot where I think you’re risky enough to merit taking a pill every day, but not so risky that I think I’m gonna give you this and undertreat your HIV that you’re about to get tomorrow.* (Shawn, Oakland provider, HIV Specialist).

### 2) Current Models of Care and Skill Sets were not Always Well Suited for Prescribing PrEP

Most providers felt that PrEP is best provided in primary care settings by providers who are comfortable working with gay men and TG women, able to discuss sexual behaviors in a non-stigmatizing manner, and are informed about HIV. Most agreed that it was important that PrEP be offered in neutral clinical locations, i.e. those whose primary purpose was not HIV treatment. Practitioners that saw positive and negative patients felt they were at an advantage for being able to provide PrEP.

*In my office stigma is not so much an issue because of my blended practice. And I do get patients who do not want to go back to the AIDS Center, they do not want to go to [local clinic] … where the clinics are kind of segregated.* (Bill, Oakland provider, PCP).

However, most providers reported that their current models of care were going to need to change to accommodate the needs of patients on PrEP, particularly with respect to adherence counseling. For those working in clinics that primarily did STI and HIV testing, this involved switching to a more longitudinal model of care.

*“[W]e are not used to having people that come back for check-ins on a regular basis… [With PrEP], we’re responsible for monitoring someone, and to make sure their kidney function is okay. That really does move into the realm of primary care. …[W]e’re going to have to do a lot of training with our own providers to make them more comfortable with doing some of that.”* (Geeta, SF provider, Community/STI Clinic).

For those in HIV clinics, providing PrEP would entail additional training to provide preventive care to uninfected individuals. One San Francisco HIV provider had serious reservations about his clinic becoming a place that offers PrEP services:

*[O]ur approach has always been to be a primary care center for people with HIV, and it would be an easy thing for us to extend that to people at risk for HIV, and assessing and prescribing, monitoring PrEP in that context.[But] I think it would be very difficult for us to wear the other hat of sort of a focused intervention where we would have like a PrEP clinic, … that would be a significant departure from our model of care.* (John, SF provider, HIV specialist).

In community-based clinics, irregular access to a medical doctor for lab monitoring and follow up was an additional concern: *“it kind of depends on whether it was something where there’s a protocol set up, where a registered nurse could provide – furnish this … we wouldn’t be able to operate it if RN’s were excluded from providing.”* (Lawrence, SF provider, Community/STI Clinic).

### 3) Providers Expressed a Need to Build Capacity to Prescribe PrEP– training, Referrals, and Establishing Reimbursement Levels for Care and Drugs

All providers noted a need to increase their clinics’ operational capacity to provide PrEP. Although low demand for PrEP was noted among the different providers’ client populations currently, all anticipated that increasing demand for the intervention would necessitate the development of screening and eligibility protocols, clarifying insurance reimbursement rates, training existing staff, and perhaps hiring additional staff. As one provider noted, “*If we wanted our medical assistants or anyone to provide PrEP, they would require some counseling training.”* (James, SF provider, Community/STI Clinic).

Public clinics with limited resources would have a more challenging time developing the billing capacity, training, and staffing infrastructure necessary to provide PrEP to their patients, and providers worried that PrEP would amplify current disparities between the public and private health systems. One provider noted:

*I think this [PrEP] is a tool for individual patients in individual circumstances. And clinics that have favorable circumstances as well, to use - kind of like – what’s the effect of bone marrow transplant gonna be on mortality rates. Not high, but if you’re one of the people who needs a bone marrow transplant, it’s awesome, and it’s there. PrEP is like that. It’s likely to exacerbate disparities rather than improve them. It’s definitely not a disparity-reducing type of intervention, because it requires a lot of infrastructure and a lot of readiness, on the part of both the patient and the clinic, to do it.* (Shawn, Oakland, HIV Specialist).

Similarly, among providers seeing patients without insurance, other support services needed to be identified and in place before doctors would consider prescribing PrEP.

*We take care of people who have histories of substance abuse, homelessness, mental illness, poverty, so getting them to be able to do something like this would require [a] belief in its importance by the providers who are struggling to get people to take meds for normal things like hypertension, diabetes, and schizophrenia. So it would require some supports, both around education, around adherence, and also financially, because we have as many uninsured as we do – we’d have to have access to meds and I’m sure it’s expensive.* (Carla, Oakland provider, Community/STI Clinic).

Public health providers also recognized the need for a community-focused education component to let people know about PrEP and its availability. This would require balancing strategies for publicizing PrEP availability widely with the need to communicate that the intervention is not intended for everyone. One idea was to try to work with social networks and popular opinion leaders to get the word out about PrEP, particularly to African American communities which are disproportionately impacted by the epidemic.

*We’ve done some work, thinking about what’s the right sort of community education part that needs to happen, who are the right kind of providers that it would be important, good to engage, what’s the right… opinion leader who has social clout…You also need to be able to educate and make this – the extent to which you can look at how to normalize some of the seeking of these types of services, or not at least have them be highly stigmatized, and how the fact that they’re related to sex, or HIV, or something… we don’t want to just put billboards up everywhere, and have everyone come and flood the system with people who are not at risk and really don’t need this intervention.* (Mary, LA provider, Public Health Official).

### 4) Concerns About Monitoring Adherence, Side Effects and Toxicities, Resistance, and Risk Compensation Among PrEP Patients

**Adherence.** Providers noted that monitoring adherence would be a challenge. Current adherence monitoring practices for HIV-positive patients, such as monitoring refills, interviewing and counseling about adherence, would need to be extended to those patients on PrEP. Several providers noted the additional burden that would create on themselves and their staff if PrEP demand were to increase dramatically. In one practice, it was envisioned that a dedicated case worker would provide the intensive adherence counseling and monitoring of PrEP patients.

Aside from demands on staff time, providers whose patient populations included young MSM and TG women expressed the need for more tailored adherence counseling to address these groups’ particular adherence challenges:

*I think a lot of young people tend to have less stable schedules… they wake up at different times during the day, they eat at different times during the day, they skip meals, their lives are just less regimented and structured and stable, and I think because of that, it’s easy to forget meds, it’s easy to - oh, I slept at my boyfriend’s house last night, so I didn’t bring my pills. Plus, they’re just less - I guess reliable in that way. They may not have their medications with them. They’re not taking them at the same time every day. They have this shame around it so they don’t want to show their friends they’re taking medication.* (Karen, Oakland provider, Community/STI Clinic).

Providers in private practice, whose patient populations were better educated and privately insured gay men, felt that episodic use of Truvada® was likely to be common among their patients. “*They might take it just 3 days before an event, if they know that they’re going to have a party or something special*,” (Wayne, LA provider, PCP). This provider felt that providers may not be able to prevent this type of intermittent use and would need to develop adherence counseling that took this practice into account.

#### Side effects and toxicities

Most providers felt that Truvada® was “generally well tolerated” and had minimal concerns about side effects. However, all providers recognized the need to carefully monitor PrEP patients for toxicities. During our discussion of Truvada® side effects, one Oakland PCP who had experience prescribing Truvada® to HIV-positive patients recalled a recent case where a patient who had been taking Truvada® for years for HIV had recently experienced acute and chronic renal failure. Another provider in Los Angeles expressed concern about the interpretation of the iPrEx trial results with respect to toxicities in particular sub-populations of patients:

*I think the fairly benign toxicity profile seen in iPrEx was at first reassuring, and then on reflection, in light of the adherence data, depressing. In that this is the toxicity that we saw during one year of treatment for people who basically took the drug less than half the time. What would the toxicity profile look like for somebody who is taking it actually according to the way it was prescribed, for perhaps a longer period of time? What would happen if we saw a less baseline healthy population, with perhaps more predisposition to renal dysfunction, like African-Americans in general?* (Hector, LA provider, HIV specialist).

#### Resistance and frequency of HIV testing

Some providers were concerned about the development of viral resistance in the event that a patient seroconverted while taking PrEP. Overall, most providers felt that quarterly follow-up with PrEP patients would be required, although some felt that monthly follow-up was required, and others that semi-annually was sufficient. Providers whose patient populations included young MSM, subustance-using MSM, and MSM of color noted that these populations generally had difficulty keeping medical appointments and might require special care in any patient tracking protocol.

*This is a pretty young MSM of color, very transient, kind of unstable population, who kind of slip in and out of care, who generally are not in care anyway. Those would be the people I would be most likely to give PrEP to, the party-hard, young MSM of color, who have high high-risk exposures and are just not able to cut down. The concern with resistance would be, let’s say if they get the PrEP, and they know how to get refills, and they fail to show up every six months for their HIV test. And then they’re still taking it, they’re still taking the PrEP off and on, not knowing they have HIV infection and not coming back to get their HIV tests.* (Karen, Oakland provider, Community/STI Clinic).

#### Risk compensation

None of the providers we interviewed were concerned about risk compensation by patients on PrEP. Overall, most providers tried to remain non-judgmental and pragmatic, while helping their patients have satisfying, yet safe, sex lives.

*I think that’s kind of paternalistic, and I try to tell my patients, these are all the strategies we have. My goal is really to make sure that people feel comfortable with that information, and that they use it in the way they can use it. I try very, very hard not to even change my facial expression when people tell me about really, really risky behaviors that they’re engaged in, and be more helpful in terms of how people navigate their lives with the tools that I can make available to them, like medications.* (Bill, Oakland, PCP).

### 5. Providers Believed in the Public Health Benefits of PrEP, Even in Light of the HPTN 052 Results

The results of the HPTN 052 trial were announced just before we went into the field for this study, and it was something that several clinicians brought up during interviews. That trial found that among serodiscordant couples where the positive partner was virally suppressed, there was a 96 percent reduction in HIV transmission to the uninfected partner [4], [25]. Given that public resources for PrEP will be limited and that HPTN 052 also showed efficacy in preventing HIV acquisition, it is important to understand provider perspectives on both biomedical prevention interventions [25], [26]. In all interviews, we found that providers saw merits in each approach, and that both should be considered essential aspects of a comprehensive HIV prevention program. One clinician researcher in San Francisco noted because some participants in HPTN 052 did become infected during the course of the study, the results of PrEP and HPTN 052 are best viewed as complementary and synergistic, and that additional modeling research could inform public health approaches that effectively utilize both interventions.

*[W]e know that there’s a sizable proportion of infections that are still occurring within partnerships, and so – and that happened within the 052 study as well, that there are a number of unlinked infections that occurred. And so only treating the positive partner isn’t going to eliminate all the infections, and so finding the right balance between treatment and PrEP I think is important to have as a target.* (Michael, San Francisco provider, Clinician Researcher).

Given the high prevalence of undiagnosed HIV infection in urban populations [27], one public health official described targeting PrEP towards higher-risk individuals as adding value to current efforts to control the epidemic through a test-and-treat approach:

*I think we’ve all recognized that if we could have an impact, there’s a real need and responsibility to really focus on making sure that we get everyone we possibly can who is living with HIV, to have an undetectable level, from a prevention standpoint… Looking at our surveillance data for 2009, 35 and 40 percent of people who are cases for L.A. County had no CD4 viral load count at all in the surveillance system, meaning they did not access care at all. And so when we know we’re dealing with that, and then we think we have about 21 percent undiagnosed, if we apply the CDC estimates to our local population. We have this pool of virus…Given that that is the setting, to the extent to which we can really focus PrEP in on the riskiest group, the tip of the iceberg, the ones that really are having just a lot of unprotected sex and a lot of risk, I think that it’s probably still needed.* (Mary, Los Angeles provider, Public Health Official).

This concern was echoed by providers working with particularly hard-hit populations, such as African American MSM [28], who saw PrEP as an essential ingredient to breaking the cycle of HIV infection.

*So if we’re aggressively treating the people who have the illness – and even a half – three-quarters – I’m sure there’s mathematical modeling that would tell us what it is – but half or three-quarters of the people at highest risk, are on – half the time, taking PrEP, then that may be the breaking point at which, when you do mess up and don’t take your pill, the person that you’re sleeping with is not going to be virally productive on that day. So there’s a point at which it starts to have an impact over a community of people. Because that’s really the way this works, is that the reason why certain communities are so impacted is because people all have sex with each other… But that’s why you can have 46 percent of the African-American gay men infected, is because there’s that hothouse concentration. And that means that if you could get enough of just that one population protected, then you could break that cycle within that community, potentially.* (Jessica, Oakland provider, HIV Specialist).

## Discussion

The iPrEx trial demonstrated PrEP efficacy for MSM and TG women, but recent studies have raised questions regarding the population-level effectiveness of PrEP in a world of limited public health resources, multiple biomedical HIV prevention approaches, and concerns surrounding appropriate implementation protocols. The recent release of the HPTN 052 trial results has bolstered policy arguments for investing scarce resources in “treatment as prevention” programs [29], perhaps to the detriment of investment in PrEP. It is in this environment that we obtained findings that we translate here into seven recommendations for PrEP to be “real-world effective”:

### Clinical Protocols and Target Populations

Prior studies have discussed the need to clarify PrEP target populations, and to establish protocols for medication dosing, adherence and side effects monitoring, and provider training in harm reduction and risk assessment counseling [12], [15]. Our study demonstrates that providers are in general agreement that PrEP should be dosed daily in line with the iPrEx trial protocol and the CDC’s interim guidance on PrEP [2], [8]. Providers believed that recipients should be monitored for side effects and possible seroconversion on at least a quarterly basis, and that clinic staff need further training in PrEP implementation. Given the central role of registered nurses in community health clinics, training programs should include protocols for clinic staff besides physicians to provide PrEP and conduct appropriate monitoring and follow up. Training protocols must therefore account for the role of different healthcare providers.

Providers were more divided on specifying PrEP target populations. Although most agreed that serodiscordant couples were ideal candidates for PrEP, there was more discussion around the worried well and individuals with multiple sex partners. While providers at publicly financed clinics were most concerned with targeting PrEP to very high-risk individuals, providers working with privately insured patients expressed a greater willingness to prescribe PrEP to any motivated patient. Given limited HIV prevention resources, existing data that PrEP is most cost-effective when targeted to high-risk individuals, as well as equity concerns, we agree that publicly financed clinics should adopt more restrictive risk-based parameters for offering PrEP. However, public health officials and providers should be aware that providers working with privately insured patients are likely to continue to prescribe PrEP to a wider pool of patients.

### Community and Provider Education

Several recent studies have called for PrEP community education to be included as part of a combination approach to HIV prevention [14], [15]. Our findings support this call, as providers reported very low demand from their patients for PrEP, even though many providers in our sample work with patients reporting very high-risk sexual behavior (e.g. multiple cases of rectal gonorrhea, repeated PEP use, low use of condoms). Also, providers believed that community education could help reduce stigma around accessing HIV-related care and prevention services. In this study, several providers spoke of partnerships between their clinics, local public health departments and community-based organizations as one effective approach for expanding PrEP community education.

Our findings also demonstrate that many providers believe they or their colleagues were in need of additional education regarding PrEP implementation. Several providers indicated that provider training in identifying appropriate PrEP candidates could help facilitate discussions with patients that reported high-risk behaviors. Non-HIV specialist providers also discussed a need for further education on adherence counseling and side effects monitoring, with STI/sexual health clinic providers in particular needing further training on long-term follow-up with patients.

### Public and Private Funding for Medication Costs

Policy analysts and researchers have identified the high cost of PrEP as one barrier to its use as an HIV prevention strategy [16], [18], [30]. As several of the providers interviewed expressed, patients privately insured through companies like Kaiser Permanente and Wellpoint have had no trouble in getting their PrEP medication costs covered [4], [19], [20]. However, providers working in publicly financed clinics held concerns that their uninsured, underinsured, or publicly insured patients would not be able to access PrEP. Moreover, public health officials in all three cities claimed that cost could be one factor in limiting access to PrEP for the most high-risk individuals, particularly in African-American and Latino communities. To prevent further disparities in HIV outcomes we recommend consistent policies for coverage of PrEP by both public and private payers of health services. Gilead’s recent decision to apply for an FDA-approved prevention label for Truvada® could help facilitate PrEP coverage for low-income individuals through state Medicaid programs and other public insurance sources [9].

### Public and Private Funding for Adherence Counseling and Monitoring

Many providers reported concerns that their patients would not be covered for adherence counseling and side effects/toxicity monitoring, and that this component of their PrEP package would be crucial for successful implementation with high-risk patients. Providers expressed that adherence counseling could constitute an additional component of a doctor or nurse visit, or could be implemented by a case manager in certain clinics. Providers interpreted side effects/toxicity monitoring generally in line with the iPrEx protocol, though several commented on the difficulty of translating certain iPrEx protocol requirements such as monthly HIV testing into their practice, instead opting for the CDC interim guidance of every 2–3 months and in some cases, every 6 months. As with medication costs, providers believed that most privately insured patients would have little trouble being reimbursed for the full PrEP package, while providers at STI/sexual health clinics, federally-qualified health centers, and other publicly financed clinics expressed more concern regarding reimbursement. As such, we recommend that providers continue to work with local public health officials to identify appropriate reimbursement mechanisms for the full array of PrEP services.

### Public and Private Funding for Social Support Services that Complement PrEP

Several providers noted that social support services would be essential to successful implementation of PrEP for high-risk individuals. In particular, providers expressed a desire to see PrEP implemented in tandem with services that address mental health, homelessness, and substance abuse. These factors were cited both as risk factors for HIV transmission and as barriers for individuals to adhere to a complex dosing and monitoring regimen like PrEP. Thus, we recommend that public health officials and providers implement and finance PrEP as part of a comprehensive package that retains funding for social support services. Such a model lies at the heart of Ryan White programs for people living with HIV/AIDS, and our findings indicate that a similar set of programs would need to be made available to many PrEP recipients to ensure effectiveness.

### STI/sexual Health Clinics, Along with HIV Specialists and Primary-care Providers, Deliver PrEP

Our study finds that STI/sexual health clinics are an obvious “first adopter” of PrEP, given their expertise in providing care to sexually high-risk individuals. In fact, PrEP demonstration projects planned to begin in San Francisco and Miami in 2012 will have STI/sexual health clinics as their service delivery sites. However, as several providers expressed, these clinics will need further training and staffing in providing long-term monitoring and care. HIV specialists and other primary care doctors will also play a crucial role in PrEP delivery. In order to successfully roll out PrEP, several challenges must be met. These include ensuring that HIV specialty practices are viewed as appropriate venues for receiving PrEP services, non-HIV specialist primary care doctors become trained in PrEP delivery, including training to increase comfort discussing sexuality and sexual health, and training on long term monitoring and adherence is provided to nurses and support staff prior to PrEP implementation.

### More Basic PrEP Research is Needed, Particularly Regarding Long-term and Intermittent Dosing

Several PrEP clinical trials assessing efficacy among populations like injection drug users and heterosexual women are underway and are scheduled to report results in the coming years. However, some researchers have called for additional basic research on PrEP efficacy, including on dosing models besides one-pill-a-day. This research would seek to address issues such as the minimum amount of time needed to achieve a therapeutic level of drug in the bloodstream and the feasibility of administering PrEP around periods of high-risk sexual behavior. Several providers in this study likewise affirmed their desire to see more basic PrEP research conducted, particularly on alternative dosing regimens. As such, we recommend that public health officials support the financing of additional studies to improve our basic clinical understanding of PrEP.

### Limitations

Our study has some limitations. First, participants represent a convenience sample that may not be generalizable to all medical and service provider populations, patient populations, and geographic settings. Demand for PrEP may change over time, and we were in the field before the trial results of Partners PrEP and TDF 2 had been widely disseminated, so it is possible that there will be more demand for PrEP in the future. We further acknowledge that most of our participants were purposively selected for inclusion in the study because of their openness to caring for TG women and MSM patients, and took great pains to be open and nonjudgmental so that their patients would feel comfortable disclosing risk behavior and possibly requesting PrEP. It is possible that other patient groups might be less willing to disclose risk to their providers, might feel less comfortable asking for PrEP, and that the patient-doctor relationship could differ in other settings. Participants were selected because they were familiar with PrEP and/or iPrEx, because they served vulnerable communities, and because another provider in the study referred them. However, our conclusions and recommendations are strengthened by the fact that those providers most likely to be experienced with the medical, fiscal, and policy implications of HIV prevention and care in the state of California were sampled. Although San Francisco, Los Angeles, and Oakland have larger epidemics than other California cities, the primary route of transmission (male to male sex) reflect epidemiological patterns apparent in other cities in California and the US, and we believe that the lessons from this research are still relevant in many settings.

### Conclusion

PrEP continues to raise questions among researchers and policymakers about how to best implement it to reduce HIV transmission among MSM and TG women. In this study, providers diverged in their definition of PrEP target populations, felt that current models of care were not always well suited for prescribing PrEP, expressed a need to build capacity, and had differing approaches on monitoring side effects and adherence. Still, most providers believed that they eventually could prescribe and monitor PrEP at their practices. STI and sexual health clinics are likely to play a key role in implementing PrEP and expanding access to individuals in our most vulnerable communities. HIV specialists and other primary-care doctors will also be called upon to provide long-term follow-up care and monitoring, and will likely continue to provide PrEP to “early adopters” particularly in cities with large gay male populations like San Francisco. Data gathered at these clinics in the coming period will be crucial to understanding the potential for PrEP to play a meaningful role in HIV prevention among MSM and TG women in the United States.
